# Catalyzing deep solid-state sulfur conversion

**DOI:** 10.1093/nsr/nwag021

**Published:** 2026-01-13

**Authors:** Peixun Xiong, Qiongqiong Lu, Ho Seok Park

**Affiliations:** School of Chemical Engineering, Sungkyunkwan University, Republic of Korea; Henan Key Laboratory of Advanced Conductor Materials, Institute of Materials, Henan Academy of Sciences, China; School of Chemical Engineering, Sungkyunkwan University, Republic of Korea; SKKU Institute of Energy Science & Technology (SIEST), Sungkyunkwan University (SKKU), Republic of Korea

**Figure 1.**
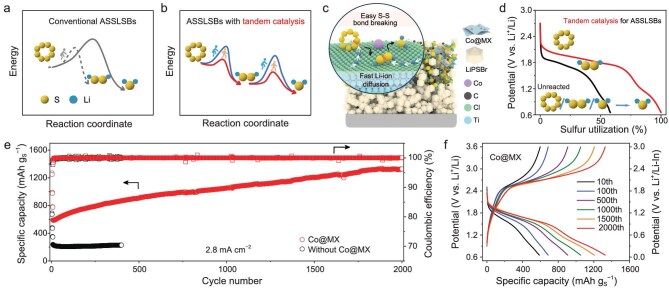
(a) Reaction pathways of conventional ASSLSBs. (b) Reaction pathways of designed ASSLSBs with tandem catalysis. (c) Schematic tandem catalytic solid-state conversion between S_8_ and Li_2_S on the Co@MX matrix. (d) Discharge processes of ASSLSBs with/without tandem catalysis. (e) Cycling performance and (f) galvanostatic charge–discharge profiles of different cycles for Co@MX-based ASSLSBs with an Li_2_S loading of 1.2 mg cm^−2^ at 2.8 mA cm^−2^. Adapted with permission from Ge *et al*. [6].

All-solid-state lithium–sulfur batteries (ASSLSBs) have attracted intense interest as a promising next-generation energy storage technology owing to their high theoretical energy density, intrinsic safety and the potential for low-cost cathode materials [1,2]. Despite these advantages, the practical performance of ASSLSBs is still limited, primarily due to incomplete sulfur conversion and sluggish solid–solid reaction kinetics [2,3]. A prevailing paradigm in the field assumes that sulfur reduction in all-solid-state systems proceeds via a direct one-step conversion from S_8_ to Li_2_S (Fig. 1a) [4]. This oversimplified view fails to resolve the severe kinetic barriers and low sulfur utilization typically observed in practice. In this context, electrocatalysts are considered as an effective pathway to accelerate sulfur redox kinetics [5]. However, catalytic studies in ASSLSBs remain rudimentary and their fundamental mechanisms have yet to be explored.

In a recent pioneering study published in *National Science Review*, Yang’s group and collaborators have demonstrated that deep sulfur conversion in ASSLSBs could be achieved through a stepwise reaction pathway enabled by tandem catalytic strategy (Fig. 1b) [6]. Instead of a direct S_8_-to-Li_2_S transformation, sulfur undergoes sequential reduction via a critical intermediate Li_2_S_2_ phase. This insight provides a fundamentally new perspective on sulfur conversion electrochemistry in ASSLSBs, highlighting that the regulation of the reaction pathway—rather than solely optimizing transport properties—is the key to unlocking the full theoretical capacity of sulfur cathodes.

To implement this tandem catalytic strategy, they designed a sulfur host comprising cobalt single-atom catalysts anchored on a conductive MXene substrate (Co@MX) (Fig. 1c and d). The atomically dispersed Co sites and the polar MXene surface perform complementary roles during sulfur conversion. Specifically, isolated Co atoms serve as highly active catalytic centers that facilitate S–S bond cleavage and sulfur activation, while the MXene substrate promotes electron transport and Li⁺ diffusion across the solid–solid interfaces. This synergistic effect significantly lowers the energy barriers for sulfur reduction and stabilizes the intermediate Li_2_S_2_ species, enabling an energetically favorable stepwise transition from S_8_ to Li_2_S.

The tandem catalysis mechanism effectively addresses two long-standing challenges in ASSLSBs: sluggish reaction kinetics and incomplete sulfur utilization. By decoupling sulfur bond dissociation and ion transport into cooperative catalytic functions, the rationally designed catalysis system led to accelerated sulfur redox kinetics without sacrificing structural stability. Consequently, the Co@MX-based ASSLSB exhibits exceptional electrochemical performance, delivering a high reversible capacity of 1329 mAh g_s_^–1^ and maintaining long-term cycling stability over 2000 cycles at a current density of 2.8 mA cm^–2^ at room temperature (Fig. 1e and f), far outperforming the catalyst-free cathode (<300 mAh g_s_^–1^). These results underscore the critical role of deep sulfur conversion in achieving high-capacity and durable ASSLSBs.

More broadly, this work establishes tandem catalysis as a general and powerful strategy for tailoring reaction pathways in all-solid-state energy storage systems. By demonstrating that sulfur conversion in ASSLSBs can be deliberately guided through intermediate states to overcome intrinsic kinetic limitations, this study advances the fundamental understanding of solid-state sulfur chemistry. The proposed approach offers new opportunities for the rational design of catalytic interfaces and may inspire the development of next-generation solid-state batteries with high energy density, long cycle life and enhanced safety.
